# Utility of sample entropy from intraoperative cerebral NIRS oximetry data in the diagnosis of postoperative cognitive improvement

**DOI:** 10.3389/fphys.2022.965768

**Published:** 2022-09-29

**Authors:** Xiaoxiao Wang, Ran Huo, Wanzhong Yuan, Huishu Yuan, Tao Wang, Nan Li

**Affiliations:** ^1^ Research Center of Clinical Epidemiology, Peking University Third Hospital, Beijing, China; ^2^ Department of Radiology, Peking University Third Hospital, Beijing, China; ^3^ Department of Neurosurgery, Peking University Third Hospital, Beijing, China

**Keywords:** cerebral oxygen saturation, postoperative cognitive changes, carotid endarterectomy (CEA), non-linear analyses, sample entropy (SampEn)

## Abstract

**Background:** Appropriate monitoring and early recognition of postoperative cognitive improvement (POCI) are essential. Near-infrared spectroscopy (NIRS) showed the predictive potential of POCI. Non-linear dynamical analysis is a powerful approach for understanding intraoperative regional cerebral oxygen saturation (rSO_2_).

**Objective:** We hypothesized that the sample entropy (SampEn) value of intraoperative rSO_2_ has the potential to predict POCI.

**Methods:** This retrospective cohort study was conducted from June 2019 and December 2020 in a tertiary hospital in Beijing, China. A total of 126 consecutive patients who underwent carotid endarterectomy (CEA) were screened. 57 patients were included in this analysis. The primary outcome was the diagnostic accuracy of rSO_2_ for the prediction of POCI.

**Results:** 33 patients (57.9%) developed POCI on postoperative day. The SampEn values of rSO_2_ were significantly higher in the POCI group (*p* < 0.05). SampEn remained an independent predictor of POCI in multivariate analysis. The area under the ROC curve (AUC) value of SampEn of rSO_2_ for POCI were 0.706 (95% CI, 0.569–0.843; *p* = 0.008). Addition of preoperative MoCA assessment and blood pressure-lowering treatment increased the AUC to 0.808 (95% CI, 0.697–0.919; *p* < 0.001).

**Conclusions:** The SampEn value of rSO_2_ showed promise as a predictor of POCI. Non-linear analysis could be used as a supplementary method for intraoperative physiological signals.

## Introduction

Carotid endarterectomy (CEA) has proven effective for the treatment of high-grade cervical carotid stenosis (Writers, 1998; Halliday et al., 2010). CEA has been associated with cognitive improvement after surgery, termed postoperative cognitive improvement (POCI) (Shi et al., 2016; Robison et al., 2019; Relander et al., 2021; Piegza et al., 2021). It has been established that POCI after CEA is related to improvement in executive functions, which may benefit from improvement in cerebral hemodynamics and associated neuronal metabolism (Relander et al., 2021). Improvements in these functions facilitate daily functioning and working abilities. Appropriate monitoring and early recognition of POCI are essential, which would prompt the scrutiny of underlying factors and promote early intervention in achieving POCI. However, validated tools for predicting POCI remain elusive.

Near-infrared spectroscopy (NIRS) showed the predictive potential of POCI. It allows for continuous and noninvasive monitoring of regional cerebral oxygen saturation (rSO_2_) (Murkin and Arango, 2009), reflecting the balance between cerebral oxygen consumption and demand. However, NIRS monitoring has not been widely used in clinical practice because of concerns regarding the diagnostic accuracy of intraoperative rSO_2_ in identifying postoperative cognitive changes (Semrau et al., 2021). The available literature (Kim et al., 2016; Holmgaard et al., 2019) has used measures to define intraoperative hypoxemia, such as a binary event based on a single rSO_2_ value or by cumulative or consecutive time with rSO_2_ under a certain threshold. More sophisticated definitions consider hypoxemia time and severity, such as the cumulative time of rSO_2_ under a threshold, area under a threshold, or time-weighted average under a threshold.

However, these definitions discard much information and therefore poorly characterize NIRS-based rSO_2_. Non-linear dynamical analysis is a powerful approach for understanding physiological signals (Henriques et al., 2020). Sample entropy (SampEn) (Richman and Moorman, 2000; Yentes et al., 2013; Delgado-Bonal and Marshak, 2019; Porta et al., 2019), a non-linear measure of regularity, has been widely applied in clinical cardiovascular studies. Greater entropy is often associated with more randomness and a lower risk of disease. We hypothesized that SampEn could be used to characterize and quantify the regularity of intraoperative rSO_2_. The major objective of this study was to estimate the entropy value of rSO_2_ for predicting the development of POCI in patients undergoing CEA.

## Methods

### Study population

We reviewed our database of patients with carotid stenosis who underwent CEA between June 2019 and December 2020. Patients eligible for inclusion were adults with symptomatic or asymptomatic unilateral moderate-to-severe carotid stenosis who were referred for CEA. The exclusion criteria were as follows: *1*) cardiogenic stroke; *2*) hemorrhagic stroke; *3*) previous vascular intervention treatment, such as CEA, stenting, clips, or coils of aneurysms; *4*) significant stenoses in the intracranial vasculature on computed tomography angiography (stenosis ≥50%); *5*) heart failure; and *6*) cerebral neoplasms. *7*) Intraoperative rSO_2_ and cognitive assessment data were missing. A total of 57 patients were identified according to the above criteria and were included in the analysis.

Baseline characteristics were collected by searching a patient database, including age, sex, height, weight, educational level, affected side, clinical symptoms, history of hypertension, hyperlipidemia, diabetes, coronary heart disease, stroke, transient ischemic attack (TIA), smoking, and drinking. Data on preoperative medication use, including antiplatelet, anticoagulant, lipid-altering, glucose-lowering, and blood pressure-lowering agent use, were also collected.

### Ethics

Ethical approval for this study (No 2019-413-02) was provided by the Medical Science Research Ethics Committee of Peking University Third Hospital, Beijing, China (Chairperson Prof Chunli Song) on 12 May 2020. The requirement for written informed consent was waived by the Ethics Committee.

### Surgical procedures

CEA was performed by the same neurosurgeon under general anesthesia using a standard approach according to hospital practice. All patients received the same anesthesia protocol. The surgeon performed deep neck dissection and vessel manipulation under a microscope. Mean arterial pressure increased by 10% before clamping. Shunting was performed if necessary. The patient was administered intravenous heparin postoperatively.

### Intraoperative rSO_2_

On the day of the surgery, rSO_2_ (FORE-SIGHT ELITE^™^; CAS Medical Systems, Branford, CT, United States) was placed on the patient’s forehead and remained in place throughout the surgery. Surgeons and nursing staff were blinded to the rSO_2_ values. All the rSO_2_ values were measured every 2 s. The rSO_2_ values collected from the affected side were used for the data analysis. Mean rSO_2_ was calculated for the intraoperative period. Minimum and maximum rSO_2_ values were defined as the lowest and highest recorded value during surgery. The rSO_2_ baseline was defined as the mean value during a 2 min period before the induction of anesthesia, while the patients were breathing room air. In addition, the relative decreases in rSO_2_ were analyzed at multiple thresholds (<baseline, <90% of baseline, and <80% of baseline). The duration below the threshold value of rSO_2_ and the area under the threshold value of rSO_2_ were calculated. The duration of all CEA surgeries differed between 61 and 157 min, with a mean surgical time of 98 min. All the rSO_2_ values were measured every 2 s. And thus, the size of the rSO_2_ time series used was between 1830 and 4710, with a mean size of 2940.

### Sample entropy

Sample entropy (SampEn) is a technique used to measure the amount of regularity and unpredictability of fluctuations over time-series data (Richman and Moorman, 2000; Yentes et al., 2013; Delgado-Bonal and Marshak, 2019; Porta et al., 2019), and to discriminate series for which clear feature recognition is difficult. It is applicable to relatively short and noisy datasets, with at least 100 data points (Richman and Moorman, 2000; Delgado-Bonal and Marshak 2019). SampEn assigns a non-negative number to a time series with larger values corresponding to greater randomness or less predictability in the data.

The SampEn algorithm is as follows (Richman and Moorman, 2000; Yentes et al., 2013). Given a sequence of numbers *u* = {*u*(1),*u*(2),…,*u*(*N*)} of length *N*, a non-negative integer *m*, with *m* ≤ *N* and a positive real number *r*, we define the blocks *x*(*i*) = {*u*(*i*),*u*(*i*+1),…,*u*(*i*+*m*−1)} and *x*(*j*) = {*u*(*j*),*u*(*j*+1),…,*u*(*j*+*m*−1)}, and calculate the distance between them as d[×(i),×(j)] = max_k = 1,2,. . .,m_(|u(i+k−1)−u(j+k−1)|). We define the total number of possible vectors by calculating for each template vector:

$$B_{i}^{m}\left(r\right)=\frac{1}{N-m-1}\times \left[number\;of\;vectors\;x_{m}\left(j\right)\;at\;a\;distance\;r\;of\;x_{m}\left(i\right),without\;allowing\;self-counting,\;where\;j=1,N-m\right]=\frac{1}{N-m-1}\times \sum_{j=1,\;j\neq i}^{N-m}\left[number\;of\;times\;that\;d\left[\left|x_{m}\left(j\right)-x_{m}\left(i\right)\right|\right]<r\right]$$ (1)

and adding all the template vectors:

$$B^{m}\left(r\right)=\frac{1}{N-m}\overset{N-m}{\underset{i=1}{\sum }}B_{i}^{m}\left(r\right)=\frac{1}{N-m-1}\frac{1}{N-m}\overset{N-m}{\underset{i=1}{\sum }}\sum_{j=1,\;j\neq i}^{N-m}\left[number\;of\;times\;that\;d[\left|x_{m}\left(j\right)-x_{m}\left(i\right)\right|]<r\right]$$ (2)

In the same way, we define the total number of matches by calculating for each model vector:

$A_{i}^{m}$ *(r) =* $\frac{1}{N-m-1}$ ×[number of vectors *x_m+1_(j)* at a distance *r* of *x_m+1_(i)*,without allowing self−counting, where

$$j=1,N-m]=\frac{1}{N-m-1}\times \sum_{j=1,\;j\neq i}^{N-m}\left[number\;of\;times\;that\;d\left[\left|x_{m+1}\left(j\right)-x_{m+1}\left(i\right)\right|\right]<r\right]$$ (3)

and adding them as: $A^{m}\left(r\right)=\frac{1}{N-m}\overset{N-m}{\underset{i=1}{\sum }}A_{i}^{m}\left(r\right)=\frac{1}{N-m-1}\frac{1}{N-m}\overset{N-m}{\underset{=i=1}{\sum }}\sum_{j=1,\;j\neq i}^{N-m}\left[number\;of\;times\;that\;d\left[\left|x_{m+1}\left(j\right)-x_{m+1}\left(i\right)\right|\right]<r\right]$

Therefore, *B_m_(r*) is the probability that two sequences are similar for *m* points, while *A_m_(r)* is the probability that two sequences are similar for *m*+1 points. Since the number of matches is always less than or equal to the number of possible vectors, the ratio *A_m_(r)*/*B_m_(r*) is a conditional probability less than unity. The parameter Sample Entropy is defined as SampEn(*m*,*r*) = lim*N*→∞{−log[*A_m_(r*)/*B_m_(r*)]}. Given N data points, we estimate this parameter by defining the statistic SampEn(*m*,*r*,*N*) = −log[*A_m_(r*)/*B_m_(r*))].

The parameters, m and r were used for calculating SampEn: m is the length of compared runs, which is suggested to have the value of m = 2, and r is effectively a filter to define the similar pattern, which equals to 0.1 and 0.25 times the SD of the original time series (Richman and Moorman, 2000). In the presence of nonlinear dynamics (Porta et al., 2019), sample entropy was calculated to characterize intraoperative rSO_2_. The surrogateTest function in R package nonlinearTseries (version 0.2.12) (Constantino A. Garcia, 2022) was used to test the presence of nonlinear dynamics of each individual rSO_2_ time-series data.

Sample entropy was calculated using EntropyHub (www.EntropyHub.xyz), an open-source toolkit for entropic time series analysis (Flood and Grimm, 2021). In the present study, we computed SampEn with m = 2 and r equal to 0.1, 0.15, 0.2 and 0.25 times the SD of the original time series, obtaining SampEn (2, 0.1, N), SampEn (2, 0.15, N), SampEn (2, 0.2, N) and SampEn (2, 0.25, N), corresponding to SampEn 01, SampEn 015, SampEn 02 and SampEn 025, respectively.

### Perioperative cognitive assessment

Cognitive performance was tested pre- and postoperatively using the Peking Union Medical College Hospital version of the Montreal Cognitive Assessment (MoCA) (Tan et al., 2021). A physician obtained the MoCA scores of the patients in a 48-h period before surgery. In addition, cognitive performance was assessed with the MoCA on three or four immediate postoperative days by the same physician. The physician was blinded to the patients’ clinical information and intraoperative rSO_2_ values. The total MoCA score was adjusted based on years of education. Add one point for an individual who has 12 years or less of formal education. Cognitive improvement was defined as at least a three-point increase in the MoCA score on the third or fourth day after surgery compared with preoperative data.

### Power analysis

A sample of 33 from the positive group and 24 from the negative group achieved 83% power to detect a difference of 0.2150 between the area under the receiver operating characteristic curve (AUC) under the null hypothesis of 0.5000 and an AUC under the alternative hypothesis of 0.7150 using a two-sided z-test at a significance level of 0.050.

### Statistical analysis

Statistical analyses were performed using SPSS version 24 (IBM Corp., Armonk, NY, United States). Categorical variables are presented as n (%). Continuous data are expressed as mean (SD) when normally distributed or as median with first and third quartiles when not normally distributed. Categorical data were compared between patients with and without POCI using a χ^2^ test or Fisher’s exact test, and continuous data were compared using an unpaired Student’s t-test or Mann–Whitney *U*-test. Baseline characteristics and intraoperative rSO_2_ parameters that were significant in the univariate analysis at a threshold of *p* < 0.1 were entered into a multivariable logistic regression model (variable selection method: Backward Likelihood Ratio). The predictive ability of SampEn for rSO_2_ was assessed using a receiver operating characteristic curve (ROC), and the AUC was calculated to assess the performance in predicting POCI. The predictive ability of SampEn, in combination with potentially influencing factors, was assessed using receiver operating characteristic (ROC) analysis and AUC values. Statistical significance was set at *p* < 0.05.

## Results

A total of 126 patients who underwent CEA at our hospital between June 2019 and December 2020 were screened. Ten patients were excluded for the following reasons: a previous history of radiotherapy to the neck area (*n* = 2), previous treatment for carotid stenosis (*n* = 3), and severe stenosis or occlusion of the ipsilateral intracranial artery (*n* = 5). Among the 116 eligible patients, 59 were excluded owing to missing data (missing clinical data, *n* = 5; missing postoperative cognitive assessment data, *n* = 11; missing NIRS monitoring data, *n* = 43). The remaining 57 patients were included in this analysis. Thirty-three (57.9%) patients developed POCI.

Table 1 shows the baseline characteristics of patients with and without POCI. The patients’ demographic characteristics were similar between the two groups. Clinical data were also similar between the groups, including the affected side, symptoms, history of hyperlipidemia, diabetes, stroke, and TIA. The proportions of self-reported smokers and drinkers did not differ between groups. The use of antiplatelet, anticoagulant, lipid-altering, and glucose-lowering drugs did not differ between groups. Patients with POCI showed worse preoperative cognitive performance on the MoCA. Moreover, the proportion of patients with hypertension, coronary heart disease, and recent use of blood pressure-lowering therapy was significantly lower in the POCI group.

**TABLE 1:** Baseline characteristics of patients with and without POCI.

Characteristics	Non-POCI (*n* = 24)	POCI (*n* = 33)	χ^2^/t	*p*
Age	65.1 ± 8.3	64.8 ± 7.1	0.159	0.875
Gender			—	1.000
Female	1 (4.2)	1 (3.0)		
Male	23 (95.8)	32 (97.0)		
Height, m	1.7 ± 0.1	1.7 ± 0.1	−0.567	0.573
Weight, kg	72.0 ± 10.6	71.5 ± 9.6	0.180	0.858
BMI, kg/m^2^	25.4 ± 3.0	25 ± 3.0	0.467	0.642
Education level			0.788	0.375
>12 years	14 (58.3)	23 (69.7)		
≤12 years	10 (41.7)	10 (30.3)		
Preoperative MoCA	20.3 ± 3.9	18.0 ± 4.3	2.087	0.042
Affected side			0.219	0.640
Left	8 (33.3)	13 (39.4)		
Right	16 (66.7)	20 (60.6)		
Symptomatic			0.805	0.370
No	1 (4.2)	5 (15.2)		
Yes	23 (95.8)	28 (84.8)		
Hypertension			4.081	0.043
No	3 (12.5)	12 (36.4)		
Yes	21 (87.5)	21 (63.6)		
Hyperlipidemia			1.050	0.306
No	7 (29.2)	14 (42.4)		
Yes	17 (70.8)	19 (57.6)		
Diabetes			0.030	0.863
No	14 (58.3)	20 (60.6)		
Yes	10 (41.7)	13 (39.4)		
Coronary heart disease			4.353	0.037
No	18 (75.0)	32 (97.0)		
Yes	6 (25.0)	1 (3.0)		
Stroke history			0.324	0.569
No	15 (62.5)	23 (69.7)		
Yes	9 (37.5)	10 (30.3)		
TIA history			0.066	0.798
No	13 (54.2)	19 (57.6)		
Yes	11 (45.8)	14 (42.4)		
Smoke history			0.244	0.621
No	8 (33.3)	9 (27.3)		
Yes	16 (66.7)	24 (72.7)		
Alcohol history			0.194	0.660
No	6 (25.0)	10 (30.3)		
Yes	18 (75.0)	23 (69.7)		
Anti-platelet therapy			0.251	0.616
No	3 (12.5)	7 (21.2)		
Yes	21 (87.5)	26 (78.8)		
Anti-coagulation therapy			—	1.000
No	24 (100)	32 (97.0)		
Yes	0 (0)	1 (3.0)		
Lipid-altering therapy			0.900	0.343
No	2 (8.3)	7 (21.2)		
Yes	22 (91.7)	26 (78.8)		
Glucose-lowering therapy			0.788	0.375
No	14 (58.3)	23 (69.7)		
Yes	10 (41.7)	10 (30.3)		
Blood pressure-lowering			7.695	0.006
No	5 (20.8)	19 (57.6)		
Yes	19 (79.2)	14 (42.4)		

POCI, postoperative cognitive improvement; BMI, body mass index; MoCA, Montreal Cognitive Assessment; TIA, transient ischemic attacks.

A comparison of intraoperative rSO_2_ between patients with and without POCI is shown in Table 2. The baseline rSO_2_ values were similar in both groups (73 vs 74%, *p* = 0.630). The mean rSO_2_ values, the lowest and highest rSO_2_ values throughout surgery, were also not significantly different between the groups. Furthermore, the duration and the area under the curve below 100, 90%, and 80% of baseline did not differ between patients with and without POCI.

**TABLE 2:** Intraoperative cerebral tissue oxygenation in patients with and without POCI.

Measures	Non-POCI (*n* = 24)	POCI (*n* = 33)	t/z	*p*
Baseline rSO2 (%)	73.4 ± 5.1	74.0 ± 4.2	−0.484	0.630
Mean rSO2 (%)	70.1 ± 4.4	71.1 ± 3.9	−0.941	0.351
Lowest rSO2 (%)	62.9 ± 5.8	63.8 ± 5.6	−0.618	0.539
Highest rSO2 (%)	78.7 ± 7.8	79.8 ± 6.4	−0.560	0.577
Mean duration of rSO2 (min)				
< baseline	70.6 (29.5, 121.1)	82.8 (0.4, 148.9)	−0.307	0.759
<90% of baseline	5.8 (0, 79.6)	1.3 (0, 85.9)	0.506	0.613
<80% of baseline	0 (0, 14.7)	0 (0, 43.3)	0.106	0.916
Area under threshold (min ×%)				
< baseline	287.3 (40.7, 1141.2)	311.6 (0.2, 1418.3)	−0.145	0.884
<90% of baseline	6.6 (0, 385.6)	1.6 (0, 643.6)	0.506	0.613
<80% of baseline	0 (0, 35.4)	0 (0, 104.7)	0.106	0.916
Sample entropy				
SampEn 01	0.262 ± 0.121	0.372 ± 0.183	−2.738	0.008
SampEn 015	0.262 ± 0.121	0.367 ± 0.189	−2.541	0.014
SampEn 02	0.251 ± 0.132	0.354 ± 0.201	−2.321	0.024
SampEn 025	0.239 ± 0.142	0.324 ± 0.217	−1.768	0.083

POCI, postoperative cognitive improvement; rSO_2_, regional cerebral oxygen saturation; SampEn, Sample entropy.

**FIGURE 1:**
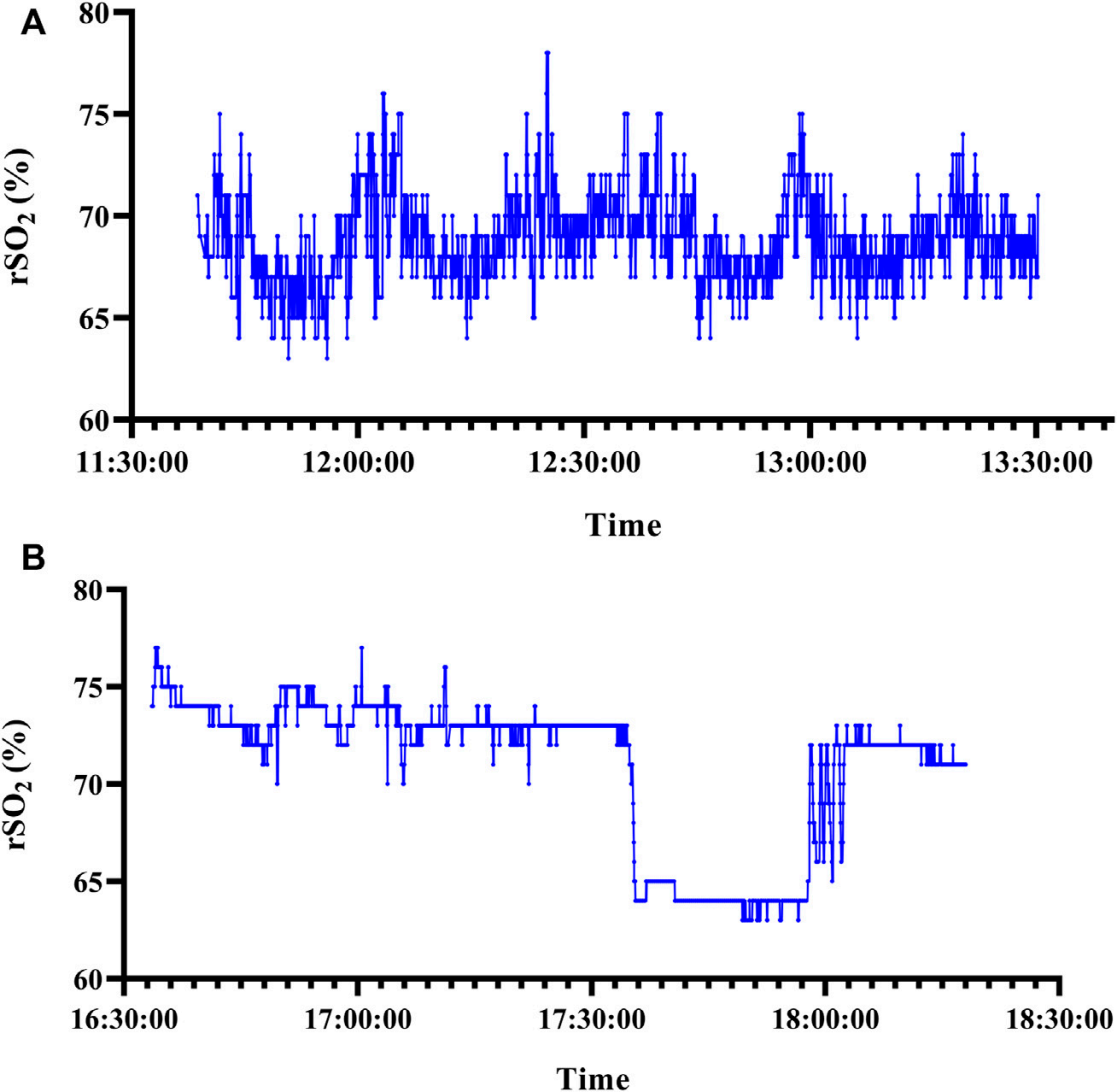
Typical tracings of rSO_2_ measured by NIRS in the POCI group. Sample entropy was 0.800. rSO_2_, regional cerebral oxygen saturation; NIRS, Near-infrared spectroscopy; POCI, postoperative cognitive improvement. Typical tracings of rSO_2_ measured by NIRS in the non-POCI group. Sample entropy was 0.123, respectively. rSO_2_, regional cerebral oxygen saturation; NIRS, Near-infrared spectroscopy; POCI, postoperative cognitive improvement.

Figures 1A,B show typical tracings of rSO_2_ measured by NIRS in the POCI and non-POCI group. Among the 57 individuals, the rSO_2_ signal of 53 subjects, including 23 (95.8%) from the non-POCI group, and 30 (90.9%) from the POCI group, showed nonlinear dynamics. The surrogate data testing rejected null hypothesis that rSO_2_ signal data of 53 subjects comes from a linear stochastic process (supplementary material). Patients with POCI had higher complexity in their rSO_2_ time series than the non-POCI group. The complexity of intraoperative rSO_2_ time series is increased, prior to the onset of POCI. As shown in Table 2, the SampEn 01, SampEn 015, and SampEn 02 values were significantly higher in the POCI group (*p* = 0.008, 0.014 and 0.024, respectively). The SampEn 025 values were higher in the POCI group, although the difference was not statistically significant. SampEn 01, SampEn 015, and SampEn 02 showed greater promise than SampEn 025. SampEn 01 was further analyzed, due to the greater difference between groups.

**FIGURE 2:**
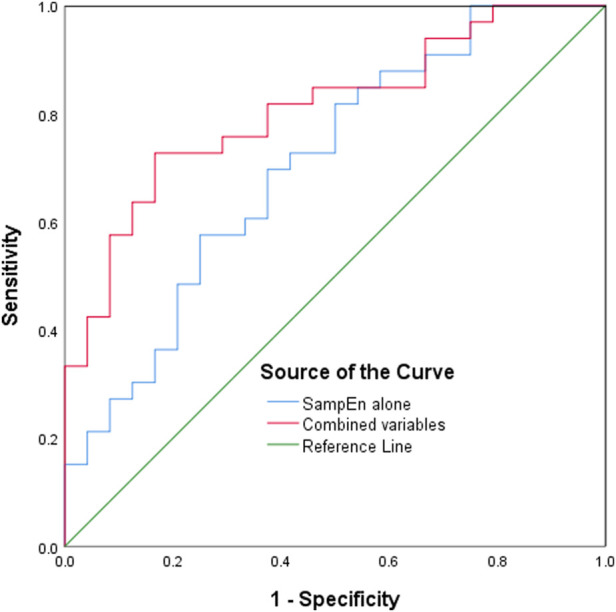
Receiver operating characteristic curves of the SampEn value of rSO_2_ signal to predict POCI. SampEn, sample entropy; rSO_2_, regional cerebral oxygen saturation; POCI, postoperative cognitive improvement.

As shown in Table 3, SampEn 01 remained an independent predictor of POCI in multivariate analysis. Figure 2 graphs the ROC curves for the SampEn 01value of rSO_2_ signal. The AUC value of SampEn 01 for POCI was 0.706 (95% CI, 0.569–0.843; *p* = 0.008), respectively. Addition of preoperative MoCA assessment and blood pressure-lowering treatment increased the AUC to 0.808 (95% CI, 0.697–0.919; *p* < 0.001).

**TABLE 3:** Predictive power of selected variables for POCI using logistic regression analysis.

	Univariate analysis		Multivariate analysis	
	OR (95% CI)	*p*	OR (95% CI)	*p*
SampEn 01 (per SD)	2.40 (1.12, 5.12)	0.024	2.44 (1.05, 5.69)	0.038
Preoperative MoCA	0.87 (0.76, 1.00)	0.047	0.86 (0.73, 1.00)	0.053
Hypertension	0.25 (0.06, 1.02)	0.053		
Coronary heart disease	0.09 (0.01, 0.84)	0.034		
Blood pressure-lowering	0.19 (0.06, 0.65)	0.008	0.13 (0.03, 0.53)	0.004

POCI, postoperative cognitive improvement; OR, odds ratio; CI, confidence interval; SampEn, Sample entropy; MoCA, Montreal Cognitive Assessment.

## Discussion

In this retrospective observational study on the diagnostic accuracy of NIRS-based rSO_2_ in predicting POCI in patients undergoing CEA, the SampEn and SampEn values of rSO_2_ showed the predictive potential of POCI. The addition of preoperative MoCA assessment and blood pressure-lowering treatment could improve the diagnostic performance.

Cognitive improvement after surgery is an important issue in perioperative medicine. Previous studies have reported cognitive benefits after CEA (Shi et al., 2016; Robison et al., 2019; Relander et al., 2021; Piegza et al., 2021). In the present study, we found that patients with preoperative better cognitive function derived greater benefit from CEA than those who had lower preoperative MoCA scores. Further study is needed to examine the mechanisms. POCI is associated with better hemodynamic parameters (Robison et al., 2019; Relander et al., 2021), suggesting that intraoperative cerebral perfusion monitoring could be a valuable predictive indicator. Several studies have reported that rSO_2_ can be used to assess cerebral perfusion and the resulting oxygenation (Kim et al., 2016; Holmgaard et al., 2019; Murkin and Arango, 2019). The available literature used baseline rSO_2_ and absolute and relative declines in rSO_2_ to establish the association between rSO_2_ and postoperative cognitive changes. However, these measures discarded much information and, therefore, poorly characterized the NIRS-based rSO_2_. Current evidence indicates that if there is a link between intraoperative rSO_2_ values and cognitive changes, better measures are needed to characterize rSO_2_ (Ortega-Loubon et al., 2019).

To the best of our knowledge, this is the first study to apply non-linear analysis to intraoperative rSO_2_. The results of the present study showed that the development of POCI was associated with a significant increase in the SampEn value of rSO_2_ signals. SampEn remained an independent predictor of POCI in multivariate analysis adjusting for preoperative cognitive function. The findings indicated that SampEn may be used to characterize intraoperative rSO_2_ and predict POCI in patients undergoing CEA. Researchers have discovered a mechanism to explain the association between the sample entropy of physiological signals and clinical outcomes. Existing studies (Pincus, 1994; Costello et al., 2020) indicate that in healthier individuals, more information processing (i.e., engagement of the regulatory components) in response to environmental challenges, such as hypoxia, is expected. As entropy is a measure of information content in complex physiological time series, evidence indicates that the entropy of physiological signals is higher in healthier individuals. Thus, patients with significantly higher SampEn values of the rSO2 signal, suggesting better cerebral perfusion and oxygenation, were more likely to be associated with better postoperative neurological outcomes.

This study has the following limitations. First, this study had a small sample size and was conducted in patients who underwent CEA at a single center in Beijing, China. Directions for future research include an association between the entropy value of rSO_2_ and postoperative cognitive changes in various clinical settings and populations. Intraoperative information was not available, including blood pressure monitoring, anesthesia, and operative procedures, which limited the exploration of the influencing factors associated with the entropy value of rSO_2_. Therefore, we did not provide potential intervention measures to achieve cognitive benefits after surgery. In addition, this study suggested that the entropy value of rSO_2_ was associated with POCI in the acute stage 4 days post-surgery. However, POCI in the stable phase, 3 months after CEA, was not assessed.

In summary, we found that the SampEn value of the rSO_2_ signal showed predictive potential for POCI. This is the first study to use SampEn to verify the diagnostic value of rSO_2_ for postoperative cognitive changes. Non-linear analysis of physical signals could be a supplementary method to perioperative indices, including NIRS-based rSO_2_. However, further work is required to test the potential value of our methodology by using a larger dataset.

## Data Availability

The raw data supporting the conclusions of this article will be made available by the authors, without undue reservation.
