# Supplementary material for: Utility of sample entropy from intraoperative cerebral NIRS oximetry data in the diagnosis of postoperative cognitive improvement
Source: Front Physiol. 2022 Sep 29;13:965768. doi: 10.3389/fphys.2022.965768 (PMC9558228; doi:10.3389/fphys.2022.965768)
Supplement: Supplementary file 1 [file Table1.DOCX]

**Surrogate data testing method**

The surrogateTest function in R package nonlinearTseries (version 0.2.12) was used to test the presence of nonlinear dynamics of each individual rSO_2_ time-series data.

This function tests the null hypothesis (H0) stating that the series is a gaussian linear process. The test is performed by generating several surrogate data according to H0 and comparing the values of a discriminating statistic between both original data and the surrogate data. If the value of the statistic is significantly different for the original series than for the surrogate set, the null hypothesis is rejected and nonlinearity assumed. For a two-sided test, 2K/α−1 surrogate data are generated. The null hypothesis is rejected if the statistic from the data gives one of the K smallest or largest values.

**Surrogate data testing results**

Among the 57 individuals, the rSO_2_ signal of 53 subjects, including 23 (95.8%) from the non-POCI group, and 30 (90.9%) from the POCI group, showed nonlinear dynamics.

The results showed that discriminating statistic for rSO_2_ signal in 53 subjects is either significantly larger or smaller than the value of the statistic for the surrogate data, and surrogate data testing rejected null hypothesis that rSO_2_ signal data comes from a linear stochastic process.

**Table S1**. Surrogate data testing results of 6 randomly selected individuals

| Group | Subjects | Surrogate data testing | |
| --- | --- | --- | --- |
| POCI |  |  |  |
|  | 2 | data.statistic < surrogates.statistics | Reject H0 |
|  | 5 | data.statistic < surrogates.statistics | Reject H0 |
|  | 7 | data.statistic > surrogates.statistics | Reject H0 |
| Non-POCI |  |  |  |
|  | 2 | data.statistic > surrogates.statistics | Reject H0 |
|  | 7 | data.statistic < surrogates.statistics | Reject H0 |
|  | 10 | data.statistic > surrogates.statistics | Reject H0 |

POCI, postoperative cognitive improvement;

data.statistic, value of the statistic for the original series;

surrogates.statistics, value of the statistic for the surrogate data;

H0, the null hypothesis (H0) stating that the series is a gaussian linear process.


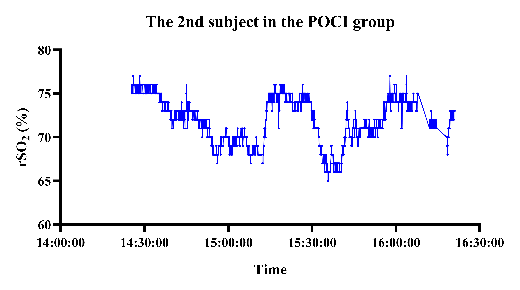

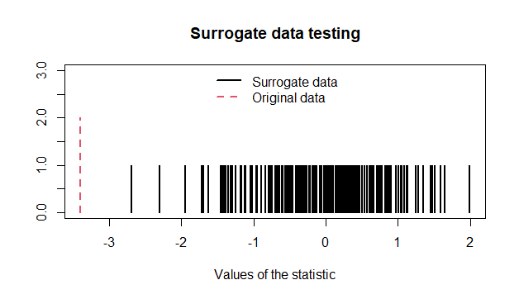


**Figure S1**. Intraoperative rSO_2_ over time (the left panel ) and corresponding surrogate data testing (the right panel) of the 2nd subject in the POCI group. In the right panel, the x-axis represents the values of a discriminating statistic for original data and the surrogate data; the y-axis represents original data or surrogate data.


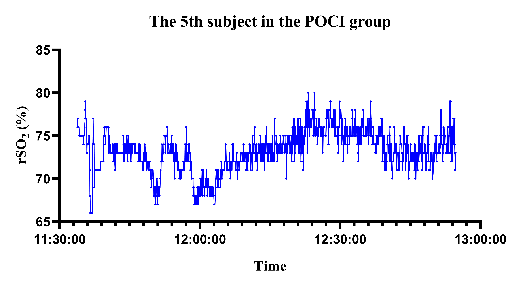

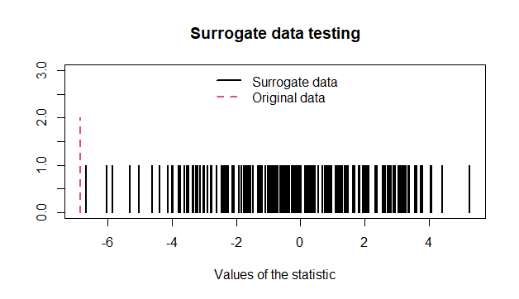


**Figure S2**. Intraoperative rSO_2_ over time (the left panel ) and corresponding surrogate data testing (the right panel) of the 5th subject in the POCI group. In the right panel, the x-axis represents the values of a discriminating statistic for original data and the surrogate data; the y-axis represents original data or surrogate data.


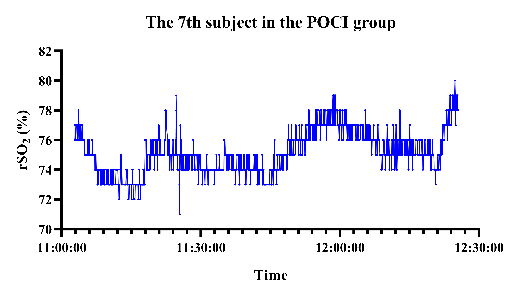

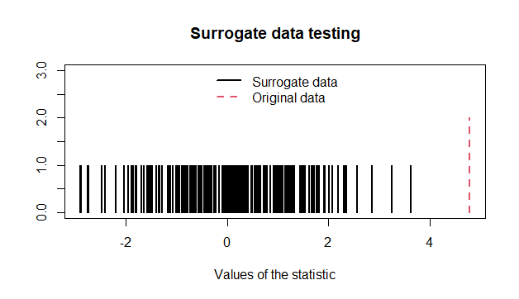


**Figure S3**. Intraoperative rSO_2_ over time (the left panel ) and corresponding surrogate data testing (the right panel) of the 7th subject in the POCI group. In the right panel, the x-axis represents the values of a discriminating statistic for original data and the surrogate data; the y-axis represents original data or surrogate data.


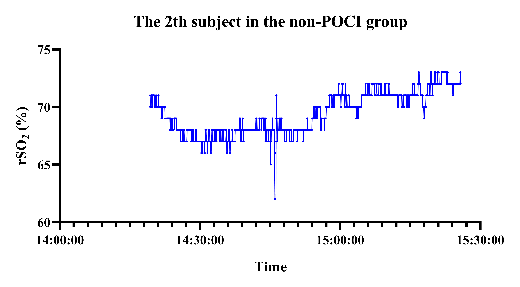

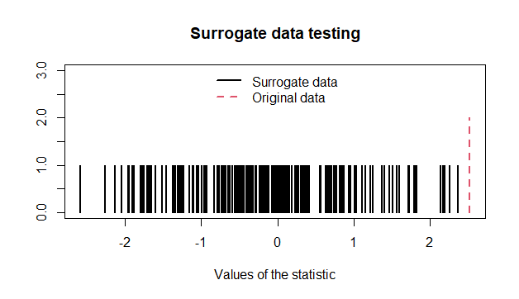


**Figure S4**. Intraoperative rSO_2_ over time (the left panel ) and corresponding surrogate data testing (the right panel) of the 2th subject in the non-POCI group. In the right panel, the x-axis represents the values of a discriminating statistic for original data and the surrogate data; the y-axis represents original data or surrogate data.


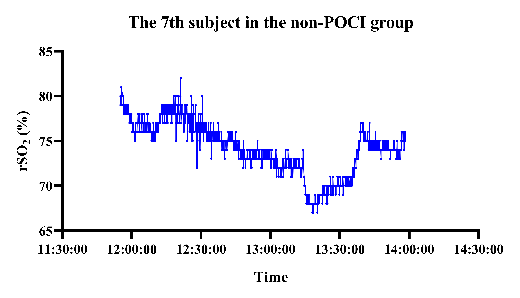

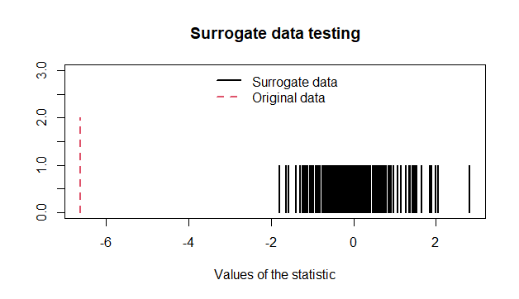


**Figure S5**. Intraoperative rSO_2_ over time (the left panel ) and corresponding surrogate data testing (the right panel) of the 7th subject in the non-POCI group. In the right panel, the x-axis represents the values of a discriminating statistic for original data and the surrogate data; the y-axis represents original data or surrogate data.


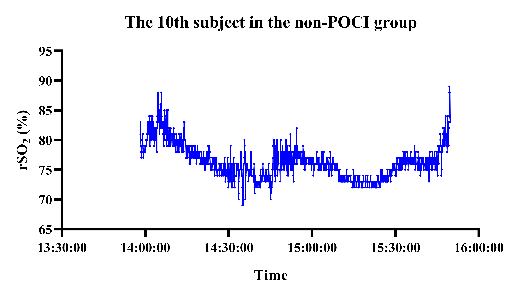

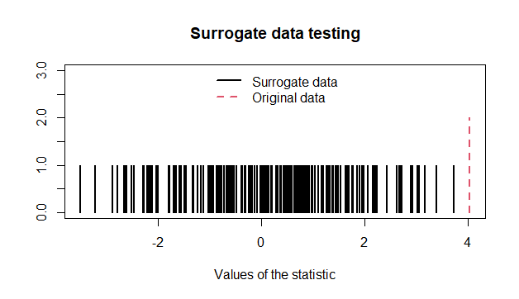


**Figure S6**. Intraoperative rSO_2_ over time (the left panel ) and corresponding surrogate data testing (the right panel) of the 10th subject in the non-POCI group. In the right panel, the x-axis represents the values of a discriminating statistic for original data and the surrogate data; the y-axis represents original data or surrogate data.
